# Acceptability and Feasibility of Using Educational Incentives for Research Participation to Advance Antiracism

**DOI:** 10.1002/eahr.60010

**Published:** 2025-07-14

**Authors:** Barbara Green‐Ajufo, Deepalika Chakravarty, Andres Maiorana, Marguerita Lightfoot, John Hamiga, Greg Rebchook

**Affiliations:** ^1^ Project policy analysis and research partnerships manager in the Division of Prevention Science at the Center for AIDS Prevention Studies at the University of California San Francisco; ^2^ Bioinformatics programmer and senior statistician in the Division of Prevention Science at the Center for AIDS Prevention Studies at the University of California San Francisco; ^3^ Specialist in the Division of Prevention Science at the Center for AIDS Prevention Studies at the University of California San Francisco; ^4^ Associate dean for research and doctoral programs in the School of Public Health at the Oregon Health and Science University‐Portland State University; ^5^ Research communication specialist in the Division of Prevention Science at the Center for AIDS Prevention Studies at the University of California San Francisco; ^6^ Professor and director of Community Engagement Core in the Division of Prevention Science at the Center for AIDS Prevention Studies at the University of California San Francisco

**Keywords:** human research ethics, research participation, research incentives, antiracism, noncash educational incentives

## Abstract

Nominal cash and gift card incentives provided to research participants have immediate financial benefits but make no lasting improvements to participants’ lives or social inequities they might experience. Our study examined the acceptability of offering a nonmonetary educational incentive as an added option to research participants as a potential to advance antiracism and address social inequities. Community members (n = 128) completed a quantitative survey; nine of whom also participated in a qualitative interview. Focus group discussions occurred with 11 researchers. Survey data were analyzed to obtain descriptive statistics. Qualitative data were analyzed using an iterative process guided by template analysis. Survey participants’ mean age was 45 years; 39% were white and 30% were Hispanic/Latinx; 80% were male; 39% had completed some college; 45% had a degree; and 71% reported previous paid participation in a research or community program. Of this group, 80% preferred cash or gift card incentives; 16% preferred an educational incentive; and 88% were likely to extremely likely to use educational incentives. Qualitative data indicated that educational incentives were acceptable but should not replace cash incentives; successful implementation would require organizational support. Noncash educational incentives may be acceptable to research participants and researchers and would help address social inequities. Successful implementation would require further research.

As part of a larger national public health antiracism agenda,^1^ an academic division (Division) within the University of California San Francisco launched an antiracism initiative paralleling the university's Antiracism Initiative^2^ to dismantle the systems, practices, and attitudes within the Division that preserved structural inequities against people of color. Research institutions are uniquely positioned to address inequities rooted in racism. The role that researchers can play to study, develop, and evaluate antiracism practices is still evolving, but key activities include dismantling structural research systems that promote racism and racial hierarchies (e.g., biased hiring, promotion practices, and mentoring processes), decentralizing whiteness in the research cycle (e.g., by creating space for diverse cultural perspectives, methodologies, and epistemologies beyond Eurocentric frameworks and prioritizing ethical standards that respect the well‐being and autonomy of participants from varied backgrounds), and improving equitable access to research funding.^3^ To further examine the role of research activities in antiracism efforts, the Division issued an internal, inclusive call to faculty, postdoctoral fellows, and staff to examine the Division's research and processes through an antiracism lens and ensure that antiracist work practices and policies were proposed, implemented, and supported.

Educational attainment is one approach to dismantling societal racism,^4^ but racism itself causes access barriers that hinder educational achievement.^5^ Our team sought to explore if and how the incentives given to research participants could serve as an antiracist tool by providing historically excluded learners with access to educational opportunities that could potentially help address structural inequities. Having more education has long been associated with multiple, far reaching, and lasting positive impacts, including healthier and longer lives, better employment options and opportunities, higher income, and improved chances for career advancement.^6^ Expanding educational opportunities for members of economically and socially marginalized communities is one kind of structural change that antiracist efforts endorse and one that research projects could actively support.

Because the U.S. HIV epidemic disproportionally affects Black/African‐American and Latinx communities,^7^ the same communities that experience educational inequities,^8^ many participants in HIV research are likely to have encountered multiple barriers to educational attainment. Identifying and implementing strategies for adult learners of color to access education, including recognizing and addressing financial barriers, is critically important to promote equity for adult education.^9^ Harrison et al. recommended providing financial incentives such as access to food and housing as a way to address racial inequities as part of a strategy to help end the U.S. HIV epidemic. Addressing educational disparities is a logical extension of this antiracist strategy and attends to its root cause.^10^

Providing incentives to research participants is ubiquitous, and research incentives have traditionally been limited to cash or gift cards.^11^ Research participants report that they support cash payments to compensate them for their time and efforts.^12^ Additionally, they generally and appropriately expect researchers to reimburse them for lost wages, and to provide transportation vouchers, food, and financial incentives as compensation for research participation.^13^ This traditional, need‐based approach to payment for participating in research is normally limited to small, noncoercive cash incentives that tend to address immediate cash needs (e.g., for bill payment, food purchase) experienced by individuals from marginalized communities who participate in research studies. Such payments are arguably needed by, and useful to, the participants, and may serve an immediate need of the participants and their families. We sought to explore the inclusion of educational incentives as an alternative or complementary option to the existing cash/gift card‐based incentive structure, providing participants with the flexibility to choose one, the other, or a combination that best met their needs.

**Educational incentives may provide learning opportunities, help explore personal interests, and develop or update skills of people who may not otherwise have the avenues or financial resources to pursue such opportunities**.

Our study aimed to assess the acceptability and feasibility of providing educational incentives to participants in research studies by assessing attitudes about educational incentives among research participants and researchers.

## STUDY METHODS

We conducted a mixed methods study with three arms: (1) a quantitative online survey of community members, (2) individual in‐depth qualitative interviews with a subset of survey respondents, and (3) small focus group discussions with university faculty and staff researchers. The study, recruitment flyers, and data collection tools were approved by the university's institutional review board (IRB).

### Recruitment

We created a flyer describing the study and incentive amounts, with a QR code and link to the online survey. We promoted the study on the Division's website, with two local HIV planning councils, on Facebook and Twitter, and at community meetings attended by large, diverse groups of local community‐based organizations (CBOs) and HIV/AIDS service providers. We encouraged people to share the flyer with their networks and constituents. To encourage participation by women, we emailed the flyer to CBOs that serve cisgender women (those whose gender identity matches their sex assigned at birth) and transgender women for distribution within their networks and at their agencies. We created a second flyer to recruit participants for the focus group discussions and distributed it via e‐mail to groups of faculty and staff. We supplemented these e‐mails with word‐of‐mouth invitations.

To be eligible for the survey, an individual had to be at least 18 years old and speak English or Spanish. For the focus group discussions, we invited university research faculty and staff study coordinators who had conducted community‐based research with marginalized communities in the local area within the last 10 years.

### Quantitative data collection

We programmed and implemented the community survey online in English and Spanish using Qualtrics. The survey included an eligibility screener and an informed consent section at the beginning. Survey questions included demographic items, history of research participation and/or participation in a CBO program, views about nonmonetary research incentives (e.g., course tuition vouchers, incentive‐type preferences, learning environment preferences, and perceived barriers to uptake of educational incentives for research participation). After eligible individuals completed the survey anonymously, they were asked if they were willing to be considered for an in‐depth individual interview via phone or Zoom and if they wanted to be considered for a random drawing for one of ten $25 gift cards for survey completion. Contact information was recorded only for participants who responded in the affirmative to either of these options.

### Qualitative data collection

From the set of community survey respondents who agreed to be considered for an in‐depth interview, we chose a purposive sample to represent a range of education levels, genders, and races/ethnicities. We conducted the individual, in‐depth, semistructured qualitative interviews averaging 30 minutes via Zoom. The interview guide included questions about reasons for participating in research, as well as perceptions and attitudes about having educational incentives added as an option for research participation and the potential impact of those incentives. We incentivized individuals to participate with a $25 gift card for their time. Interviews were audio recorded and transcribed using Otter.ai software. Research staff reviewed the transcripts for accuracy and made corrections when warranted, wrote field notes after each interview, and met throughout the data collection period to identify emerging impressions.

The focus group discussions with research faculty and study coordinators followed a semistructured interview guide to elicit views about the existing monetary research incentive structure and its effects on research participation, the proposed nonmonetary educational incentive, its anticipated effect on the lives of research participants, and its feasibility in the field. Focus group discussions were conducted via Zoom; they lasted an average of 54 minutes and there was no monetary incentive provided. Two of our study team members conducted the interviews and focus group discussions. They wrote detailed field notes following each focus group discussion and met throughout the period of data collection to compare notes and identify emerging themes.

### Data analysis

We analyzed the online survey data by generating descriptive statistics for the overall sample and then calculated frequencies and measures of central tendency for questions of interest.

For the qualitative data, we followed an iterative analysis process guided by template analysis,^14^ a type of thematic analysis, as a flexible technique to organize data, develop conceptual themes, and present the perspectives of participants in the interviews and focus group discussions. First, a lead analyst summarized each interview and focus group discussion transcript using field notes as a reference and the summary was reviewed by the second analyst. Second, using a cross‐section of the interviews and focus group discussion transcripts, the two analysts developed and refined a list of domains based on the study goals, interview questions, and themes emerging from those transcripts. Next, the analysts organized the preliminary domains into matrices to allow the data to be easily visualized, and as they continued with the analysis and populated the matrices, they arrived at a final list of domains. The final domains from the individual interviews were (1) background, (2) participation in previous studies, (3) opinions about educational incentives, (4) impact of educational incentives, and (5) other comments. The final domains from the focus group discussions were (1) opinions about noncash educational incentives, (2) how to implement noncash educational incentives, (3) the potential impact of noncash educational incentives on research workflow, (4) impact of noncash educational incentives on research, and (5) theoretical issues related to research incentives. During the data analysis process, the analysts met to discuss and resolve any discrepancies in data interpretation. Finally, they examined the matrices to identify salient themes that emerged across various conceptual categories related to the acceptability and feasibility of nonmonetary educational research incentives.

## ONLINE SURVEY RESULTS

We received responses to the online survey from 128 community members. Respondents had a median age of 45 years and were racially and ethnically diverse with 39% identifying as white; 30% as Hispanic/Latinx; 13% as Black/African American; 9% as Asian; and 9% as either multiracial, American Indian/Alaska Native, Native Hawaiian/Other Pacific Islander, or other (see table 1, which is available online; information about accessing this material is in the “Supporting Information” section at the end of this article). The majority (80%) were male; 13% were female; and 7% identified as transgender, nonbinary, or other. The sample was also diverse in educational attainment, with 16% having completed high school/GED or less, 39% having completed some college, and 45% having a college degree. A fifth of the sample had no prior exposure to either participating in research or receiving services at a CBO; 41% had prior exposure to only research studies, 10% to only CBOs, and 29% to both research studies and CBOs. Further, 71% reported being a paid research or CBO participant in the past.

Participants reported diverse views, preferences, and barriers regarding educational research incentives (see table 2 online). An overwhelming majority (89%) affirmed that completing a course on a topic that they were interested in or passionate about was a path to enriching themselves, their family, and/or their community. However, when asked to consider a scenario where they would participate in a research study requiring them to spend an hour answering questions in a survey or an interview, a large majority (80%) picked the $40‐$50 in cash or a gift card as their most likely choice of research incentive, while 16% picked an education incentive (i.e., a coupon worth $100‐$150 to enroll in a course of their choosing) as their most likely choice. However, most participants (n = 113, 88%) said they would take advantage of an educational incentive that allowed them to enroll in a course of their choosing, answering: extremely likely (n = 31, 24%), very likely (n = 26, 20%), likely (n = 21, 16%), and somewhat likely (n = 35, 27%). Only 12% (n = 15) were *not at all likely* to utilize an educational incentive. Further analysis revealed that, of these 15 participants, 12 already had a college (bachelor's or master's) degree.

Participants who were open to utilizing an educational incentive (n = 113) were further queried about those incentives. The majority (n = 80, 71%) preferred an online course while the remaining (n = 33, 29%) preferred in‐person courses. Community colleges were ranked as the first choice of location by approximately a third (33%) of the respondents and university extension programs were the first choice for a quarter (25%) of the respondents. When queried about reasons that might prevent them from taking a course if given an educational incentive (participants could check all that apply), nearly half (48%) responded that they did not anticipate any barriers. The remaining participants’ perceived barriers included lack of time (27%), a feeling of nervousness/discomfort with enrolling in a course (14%), beliefs about not doing well/succeeding/not belonging in a course (10%), lack of interest in further learning (6%), and transportation hurdles (3%).

### Individual interviews

We conducted individual interviews with nine community members who had completed the survey: seven cisgender men (four Hispanic/Latinx, two Black/African American, and one multiracial—Asian, Native Hawaiian/other Pacific Islander, and white) and two Black/African‐American cisgender women (see table 1). Interview participants’ median age was 50. Two interviews were conducted in Spanish and the remaining were in English.

### Research motivations

Reasons for participating in research included altruism; curiosity; interest in science; community advocacy; the potential benefit of an intervention, test, or medicines; and financial need. These reasons were not strictly exclusive of each other; the extent of overlap depended on the participant's personal profile (e.g., education level, financial situation, and previous experiences with research). Five individuals who participated in the qualitative interviews reported having had a history of research participation on their quantitative survey; the other four participants reported having no history. However, all nine participants in the qualitative interviews reported having a history of participation in research. Four of the nine participants connected their present or past financial situation to reasons for participating in research; the other five indicated that receiving a monetary incentive for their participation in research was a perk they did not necessarily need but could use to buy something they wanted.

### Acceptability of educational incentives

All interview participants indicated that educational research incentives toward a course of their choice would be acceptable. They stated that such educational incentives may provide learning opportunities, help explore personal interests, and develop or update skills of people who may not otherwise have the avenues or financial resources to pursue such opportunities. They mentioned that facilitating access to, or breaking down barriers to, continuing education may allow people to have a different vision of themselves, advance their life in new directions, and contribute to self‐empowerment and self‐improvement. Additionally, two participants emphasized that providing educational incentives to research participants would be well‐received in the current socioeconomic environment, since people were eager to pursue new opportunities such as jobs and education after the Covid‐19 pandemic.

Referring to their support of nonmonetary educational incentives (quotes from the community interviews use pseudonyms to protect the participants’ identities), Johnnie (age 60, Black/African‐American male) said: “If you can help a man, better to teach them how to fish, [that] can last a lifetime, instead of giving them fish to eat a meal for that night.” Diego (age 50, Latino male) also referred to the same proverb to illustrate the potential benefits of providing educational incentives that potentially could initiate a life changing experience that has a lasting impact, instead of providing monetary incentives to be used for immediate needs. He stated: “Because 25 bucks to I don't know, California Kitchen is great. But to offer something [a course] that I'm going to learn and use and benefit from in the long run for longer time. That's even better. The idea is to empower the body, mind, and spirit. I think it's genius.” Tommy (age 32, Black/African‐American male) considered the opportunity to take a course as something positive to focus on that could facilitate exposure to new ideas, and different people and environments. For him, access to further educational opportunities would help, as he stated, “make the ground equal for everybody, because we're learning together. That's going to be like our unique little relationship. That's an experience we'll be able to say that we've shared together.”

### Incentive variety

Interviewees mentioned that people who participate in research are used to receiving a monetary incentive and use the money to take care of financial needs (e.g., buy food, help pay for utilities, etc.), and that they would need time and education to see the value of an educational incentive. Diego said, “It's the American way, we've learned instant gratification. Our muscles are trained to accept quick things, quick fixes, rarely hearing of somebody saying, ‘Hey, I'm going to offer you a workshop or class or training’, something to empower you.” He added that it may take some time and retraining for people to accept the concept of educational incentives. Johnnie expressed the same sentiment, saying that “the precedent giving gift cards, it's gonna be hard to wean people off of that idea.” Interviewees indicated that educational incentives would need to be offered as one choice among others, including cash or gift cards, since educational incentives may not be appropriate for everyone. Interviewees and researchers in the focus group discussions suggested various other incentive options, discussed below (see table 3).

**Table 3:** Research Incentive Options Suggested by Study Participants

Community interviewees	Researchers
Cash Gift cards Vocational training/skills building Cultural events/experiences (concerts/ museums, dinners) to which people would not normally go/have access to, and beyond their comfort zone or neighborhood Record expungement School tuition/books	Cash/smartphone remote cash payment Gift cards Employment, skills building, resume writing, job training, and placement Bridging incentives with skill‐building interventions Referrals to food bank, other services, or to CBOs

### Concerns and barriers

Some interviewees mentioned concerns or barriers to the acceptability of educational incentives, including the need for a monetary incentive to help cover basic needs superseding any interest in educational incentives. They reiterated other reasons for a lack of interest in educational incentives, including health issues, already being in school, lack of time, course length, feeling intimidated by the process of taking a course, not knowing what course to choose, and discomfort taking a course or being on a campus; most of these reasons were also mentioned in the online surveys. Johnnie stated he was the type of person that would welcome educational incentives because he now, after overcoming drug addiction, had a good life and good health, but acceptance would not be the case for some people in his community who are, as he said, “stuck in day‐to‐day life.” He said, for some people in his community, “research is looked at as that extra benefit. Lot of people do it for altruism, but in the Black community with economics the way it is, they can't afford to be altruistic.” They see research [monetary incentive] as “a chance to supplement their income, supplement their life.”

### Institutional assistance/navigation

Johnnie explained that registering for, choosing, and enrolling in a course may be confusing or intimidating for some who would need and benefit from assistance to move through the process: “It wouldn't be so scary if they know they have someone that's going to be by their side.” He added that, while it may be a slow process, if implemented correctly, eventually people in the community would become ambassadors and through word of mouth, promote the acceptance of educational incentives. In fact, another participant, Rose (age 51, Black/African‐American female), was an example of someone who would benefit from assistance with registering for a course. She participated in research and marketing studies for the financial incentives, but also stated that she was looking for educational opportunities to start a new cycle of self‐improvement. Rose had tried to enroll in a psychology course but found the process complicated. She said, “I'm trying to stop the cycle and start a new one [self‐improvement]. That [meeting people] would be the main reward. I want to meet different people that aren't drinking or drugging and doing something with their life, you know, like going to school or whatnot. I'm open to new ideas and new concepts and new theories. I did register for San Francisco City College. But it was confusing. After registration, I didn't know what next step to take. I probably should have taken the initiative to speak to a counselor. But it stopped me because it was too complicated. And a laptop would have been better because I'm trying to do everything from my phone.”

### Improving race relations

Interviewees were asked if offering educational incentives is a way to introduce learning opportunities to those who might not have them, and if taking courses would put in proximity people from different racial backgrounds through course activity and would result in interactions that would help them have better racial relationships. All the participants who responded indicated that being in learning situations with people from different racial backgrounds who would not normally be in their life would potentially help build and improve racial relationships. Jimmy (age 56, multiracial [Asian, Native Hawaiian/Other Pacific Islander, white] male) elaborated that access to educational opportunities (taking a course) would help one meet different people and have their life affected in several ways, saying, “you know, expose you to people that you get along with. And, and it also furthered friendships, because sometimes you would see the same people in different classes.” Similarly, when asked if taking a course put people in situations that allowed them to meet people from different race/ethnic groups and other demographics and be better human beings toward each other, Tommy agreed that it would.

## FOCUS GROUP DISCUSSIONS WITH RESEARCHERS

We conducted three focus group discussions with 11 early‐, mid‐, and advanced‐career university researchers, including faculty and research coordinators. Participants included eight cisgender women, one transgender woman, and two cisgender men. They represented different race/ethnic groups: five white, one Black/African American, two Latinx, two Asian, and one other). We report the primary themes from the focus group discussions below.

### Acceptability of educational incentives/variety of incentive types

Researchers expressed some of the same opinions as the community interview participants—stating that educational incentives were acceptable to provide to participants and that they would need to be provided in conjunction with other incentive options, including monetary ones, because educational incentives may not be appropriate for everyone. Referring to the need for monetary incentives, some researchers pointed out that for disadvantaged, historically marginalized populations, cash incentives address a direct need, regardless of whether researchers and funders endorse how the monetary incentives are used, with one researcher stating: “Even if they want the option of being able to take a class or some supplementary education, they realistically don't have that option. They need a short‐term immediate solution” (Researcher 1). Researcher 2 passionately expressed their perspective, saying, “I love what you're talking about [educational incentives]. Everyone deserves to learn more and to elevate and to be exposed to different ideas and different lives. That's how we can transcend. That's how we dream and see beyond our harsh realities. I don't know if education is the only way. If you offer education to someone who hasn't taken his meds that day, because he hasn't eaten, that's [adding] insult to injury.”

### Educational incentives would position research in a framework of antiracism, equity, and social justice

Researchers expressed that providing incentive options—an element that has traditionally not been present in research—including monetary and educational ones, would better address and help reconcile the needs of diverse populations and the diverse reasons for their participation in research. Furthermore, some added that providing incentive options would help situate research within an antiracism, equity, and social justice framework. As Researcher 3 explained, “[If] we present [incentive] options, that seems like the most viable and makes the most sense, because we don't want to pigeonhole participants into getting just one type of incentive.” Most of the researchers mentioned that providing incentive options would be a way of accepting the reality of people's lives, reflecting the most beneficial or least exploitative way to engage people in research. Some pointed out that financial incentives that address an immediate need may be what first attracts people to participate in research, and to then become interested and engaged in a project. According to Researcher 4, “Wanting to participate in research and making money from it are not mutually exclusive. There's plenty of people who may come into research for the money and in their last five minutes of the interview or survey express their gratitude for a particular project. Initially, they may come in because of the incentive, and really leave [with] interest in the project.”

### Far reaching positive consequences of educational incentives

Some researchers saw providing different incentive options as an opportunity to revisit the current research incentive structure and reexamine the ethical principles of beneficence, respect, and justice underlying the protection of human subjects, principles that assume people are all equal and participate in research for the same reasons. Incentive options may contribute to upending what one researcher referred to as a “research economy,” in which some people only participate in research because of the monetary incentives.

### Incentive options

The researchers indicated that an incentive structure that includes options may help boost participants’ self‐esteem, increase skills, provide resources, and perhaps contribute to individual empowerment as well as engagement in research (see table 3). One researcher stated, “So, their engagement in research is different because it's [other incentive option] not just like short term cash, but it's something that's gonna like stay with them for longer” (Researcher 5). They also perceived that such a structure would better align with the goals of community‐engaged research; it could help to restore trust and combat skepticism and potential feelings of exploitation related to research. Researchers mentioned that incentive options would need to be selected utilizing a community‐driven process, perhaps through community advisory boards.

### Need for centralized institutional support and infrastructure

While researchers were enthusiastic about restructuring incentives, they expressed concern regarding the feasibility. Researchers in two of the focus group discussions emphasized that restructuring research incentives to provide options would only be feasible with the presence of a dedicated university‐wide system of resources. They explained that it would be a “heavy lift” for individual research studies to design their own multi‐option incentive structure; it would also overburden research staff if they had to assist participants in navigating those options. Researchers envisioned a centralized university system—a study participant and university community relations office with dedicated staff to provide guidance about incentive options, resources, and referrals. This office would navigate or guide participants through the process of selecting an incentive option, and, depending on the chosen option, perhaps even provide coaching and mentoring to help them best utilize it.

### Process recommendations for success of the alternative incentive structure

Researchers were also aware of the many steps that would be required to implement a new incentive structure including buy‐in from university leadership and IRB approval of the structure, and the need to educate funders about restructuring incentives. They cautioned (1) about the need to start small with the restructuring, (2) that current funding for research studies could not accommodate any additional costs of an educational incentive structure, and (3) most importantly, to not further perpetuate inequities by creating another system that people with resources are more likely to take advantage of and benefit from, rather than benefitting those who do not have the resources.

## DISCUSSION

The results of this study are sufficiently encouraging to warrant further investigation into the idea that educational incentives may be acceptable to both participants in human subjects research and those conducting the research. Very few participants said that they would not take advantage of an educational incentive if one were offered, and most of those participants already had completed a college degree and likely felt that they had little to gain from the proposed incentive. While monetary incentives for research participation were still the number one incentive choice among community members, well over three‐quarters of the community members surveyed said that educational incentives were a positive path to enriching themselves. Community members and researchers both agreed that educational incentives could further social justice and antiracism efforts, but both groups also pointed out potential difficulties in implementing a research compensation structure that included educational incentives such as the need for additional support and research infrastructure enhancements. Additional funding would certainly be necessary to offer meaningful educational incentives. Efforts to add educational incentives to current research compensation systems cannot exist in a vacuum.

In 2020, an antiracism working group was convened in our Division and charged with identifying priorities to promote a more antiracist climate in the Division. Among multiple activities, the working group conducted a Division‐wide antiracism climate survey and a mini‐retreat. The survey aimed to understand how racism was manifesting within the Division, including whether and how Division members were experiencing racism and microaggressions, with the goal of informing positive, proactive steps toward advancing a culture and practices to enhance racial equity based on the findings. The mini‐retreat prioritized these top two areas for attention: (1) addressing white‐centric norms and (2) conducting research on racism and antiracism. The work of this study on educational incentives aligns with these priority areas and confronts the historical norm of paying research participants a small amount to encourage their participation without being coercive. While much social/behavioral research benefits participants indirectly by contributing to *long‐term* efforts that increase health equity and by addressing disparities, or by facilitating positive changes in the community, the *immediate* impact of research participation on the lives of the participants is often negligible.

While providing educational incentives to at least partially pay for the cost of taking a single class is not going to achieve educational equity, opening the door to educational opportunities may stimulate the desire to pursue additional education by illuminating a previously unconsidered possibility. As Edelman said, “you can't be what you can't see,” emphasizing the need for and value of representation, and the impact of role models and opportunities in shaping young peoples’ aspirations.^15^ Therefore, individuals from socially, economically, and racially marginalized populations who do not routinely see images of themselves in college or post‐high school learning environments in everyday life or the media may not have any impetus to seek higher education—especially when they may have to overcome barriers constructed by systemic racism to do so.

We contend that adding an educational incentive to the current cash/gift card incentive structure may serve as a catalyst to introduce research participants, particularly those from marginalized races/ethnicities, to rewarding learning experiences that ultimately could become a pursuit. We also contend that educational research incentives could become a powerful antiracist tool that buttresses exposure to adult educational and development opportunities aimed to empower and build community. Creating opportunities for historically excluded learners to access educational resources that they may see as out of their reach is antiracist at its core because access to educational options can theoretically crack open a door to social and health equity. If research participants have the option of accessing courses and other learning opportunities, either instead of or in addition to cash payments, it may help reduce barriers created by systemic racism and produce new opportunities for personal growth. Finally, educational settings assemble people from different racial/ethnic backgrounds, providing the potential to establish cross racial/ethnic relations and incubate antiracist behaviors, beliefs, and actions at the individual level.

### Limitations

Assessing acceptability and feasibility of hypothetical educational research incentives was exploratory in nature, and the attitudes expressed about educational incentives may not match actual behavior if educational research incentives become available. Pilot testing is necessary to see if research participants would choose an educational incentive if offered and to determine the kind of logistical support necessary to put educational incentives into practice. Additionally, our survey was conducted online, potentially biasing the results toward those who are more technologically savvy. Finally, our sample size was small and limited to a small geographic region, possibly limiting its generalizability to broader populations and other regions.

### Future directions

With this documented interest from research participants and researchers in adding the option of educational research incentives to the current cash/gift card incentive structure, future inquiry is needed to determine obstacles and how such a mechanism could be implemented. The cost of doing research has increased over the past decades, but the funding limits on most research grants has not increased to keep pace with the cost increase (e.g., the cap on the National Institutes of Health R01 funding mechanism has not increased in many years). Due to rising research costs and limited funding for participant incentives, many studies are unlikely to provide incentive payments at the proposed voucher value of $100‐$150, which is obviously less than the cost of most courses. Therefore, the next step for researchers is to address this gap and explore the availability of institutional support, which would be central for this alternative mechanism's success.

To enhance the feasibility and success of educational research incentives, it will be important to engage community colleges and other continuing education providers. Thus, an additional step that could influence the success of educational research incentives would be to collaborate with these institutions to identify ways for them to effectively partner with research organizations and support the community by, for example, subsidizing course costs or offering other supportive measures. We plan to conduct additional formative research to determine if partnerships can be built between academic institutions and philanthropic organizations and/or corporations to fund and implement educational research incentives.

Furthermore, additional research is also needed to determine the kinds of educational incentives (e.g., partial tuition vouchers, microgrants,^16^ gift certificates, coupons) that would be most acceptable to research participants. The logistics necessary to issue and redeem these incentives also needs to be determined.

The long‐term effects of incorporating educational incentives into the existing cash/gift card incentive structure remain uncertain and warrant further exploration. We hope to pilot test this innovative approach and, if successful, collaborate with key partners to fully integrate and sustain education‐based research incentive options. Over time, longitudinal data will help assess whether this approach can achieve meaningful quality‐of‐life improvements for research participants from marginalized populations and whether it aligns with and enhances research institutions’ antiracist frameworks.

## ACKNOWLEDGMENTS

This research was supported by Division of Prevention Science funds allotted to finance small antiracism projects and by the Center for AIDS Prevention Studies Center Grant. We gratefully acknowledge the contributions of 128 community members and 11 academic researchers who generously shared their time and insights by completing a quantitative survey and/or participating in qualitative interviews. Their participation was essential to the success of this research.

## Supporting information

Tables 1 and 2 are available in the “Supporting Information” section for the online version of this article and via *Ethics & Human Research*'s “Supporting Information” page: https://www.thehastingscenter.org/supporting-information-ehr/.

Supporting information

Supporting information
