# Supplementary material for: Acceptability and Feasibility of Using Educational Incentives for Research Participation to Advance Antiracism
Source: Ethics Hum Res. 2025 Jul 14;47(4):18–28. doi: 10.1002/eahr.60010 (PMC12258617; doi:10.1002/eahr.60010)
Supplement: Supplementary file 1 — Supporting information [file EAHR-47-18-s001.pdf]

E&HR

Acceptability and Feasibility of Using Educational Incentives for Research Participation to Advance Antiracism

BARBARA GREEN-AJUFO, DEEPALIKA CHAKRAVARTY, ANDRES MAIORANA, MARGUERITA LIGHTFOOT, JOHN HAMIGA, AND GREG REBCHOOK

Table 1: Sample Characteristics of Community Participants in the Surveys and Interviews

**Characteristics**	**Online community survey (N = 128)**		**Individual interviews (N = 9)**	
Age (years)–median (range)	45	(21-89)	50	(32-60)
	**n**	**%**	**n**	**%**
Race-Ethnicity				
White	50	39%	-	-
Hispanic/Latinx	39	30%	4	44%
Black/African American	16	13%	4	44%
Asian	11	9%	-	-
Multiracial (n = 5), Other (n = 3), AI/AN (n = 2), NH/PI (n = 2)	12	9%	1	11%

Gender				
Male	103	80%	7	78%
Female	16	13%	2	22%
Transgender, nonbinary, other	9	7%	-	-
Education				
High School/GED or less	21	16%	2	22%
Some college	50	39%	6	67%
Bachelor's or master's degree	57	45%	1	11%
Prior exposure				
Research study only	52	41%	4	44%
CBO only	13	10%	-	-
Both research study and CBO	37	29%	1	11%
Neither research study nor CBO	26	20%	4	44%
History of paid participation in research studies or CBO programs	91	71%	5	56%

Note: AI/AN—American Indian/Alaska Native, NH/PI—Native Hawaiian/Other Pacific Islander
