# Supplementary material for: Acceptability and Feasibility of Using Educational Incentives for Research Participation to Advance Antiracism
Source: Ethics Hum Res. 2025 Jul 14;47(4):18–28. doi: 10.1002/eahr.60010 (PMC12258617; doi:10.1002/eahr.60010)
Supplement: Supplementary file 2 — Supporting information [file EAHR-47-18-s002.pdf]

E&HR

Acceptability and Feasibility of Using Educational Incentives for Research Participation to Advance Antiracism

BARBARA GREEN-AJUFO, DEEPALIKA CHAKRAVARTY, ANDRES MAIORANA, MARGUERITA LIGHTFOOT, JOHN HAMIGA, AND GREG REBCHOOK

Table 2: Responses from Community Survey Participants (N = 128) about Educational Incentives

	n	%
Viewed educational incentives as a path to enriching themselves, their family, and/or their community	114	89%
Preference for various incentive options		
$40-$50 in cash or gift card	103	80%
A coupon worth $100-$150 to enroll in a course of choice	20	16%
$10 in cash AND a coupon worth $75 to use as partial payment toward a course of choice	4	3%
All options work	1	0.8%

Likelihood of taking advantage of educational incentive if offered		
Extremely likely	31	24%
Very likely	26	20%
Likely	21	16%
Somewhat likely	35	27%
Not at all likely	15	12%

Among those extremely, very likely, or somewhat likely to take advantage of educational incentive (n = 113):

Preferred course mode		
Online	80	71%
In-person	33	29%
Top-ranked choice of places to take a course[1]		
Community college	37	33%
University extension program	28	25%
Barriers to taking a course if educational incentive is offered [2]		
None	54	48%
Lack of time	30	27%
Nervousness/discomfort with enrolling in a course	16	14%
Belief about not doing well/succeeding/not belonging	11	10%

No interest in further learning	7	6%
Transportation hurdles	3	3%

Interview results:

[1]. From five options: Community college, trade or vocational school, university extension program, other adult education programs, and other.

[2]. Respondents who did not check ‘None’, could check all that applied from ten additional provided options (categories have been collapsed for reporting).
